# Health-Related Quality of Life Deterioration Among Tuberculosis Patients During the COVID-19 Pandemic: a Convergent Mixed-Methods Study of Syndemic Vulnerability in Urban Bangladesh

**DOI:** 10.1007/s44197-026-00580-5

**Published:** 2026-05-25

**Authors:** Santu Das, Rantu Das

**Affiliations:** 1 Department of Economics, Jahangirnagar University, Savar, Dhaka, 1342 Bangladesh; 2 Department of Public Health and Informatics, Jahangirnagar University, Savar, Dhaka, 1342 Bangladesh

**Keywords:** Tuberculosis, COVID-19, Health-related quality of life, EQ-5D-3L, Syndemic, Bangladesh, Mixed methods, Pandemic preparedness, DOTS, Urban health

## Abstract

**Background:**

United Nations Sustainable Development Goal 3 (Target 3.3) calls for ending the tuberculosis (TB) epidemic by 2030; yet TB programmes in low-income settings remain acutely vulnerable to pandemic disruption. During the COVID-19 first wave, TB patients in urban Bangladesh faced a convergence of disease burden, pandemic-related stressors, and poverty-driven resource constraints. Systematic evidence quantifying health-related quality of life (HRQoL) deterioration in this population and identifying its modifiable structural determinants is limited.

**Methods:**

A convergent parallel mixed-methods study was conducted among 439 TB patients attending 12 Directly Observed Treatment, Short-course (DOTS) clinics across six municipal districts in Dhaka, Bangladesh, between August and September 2020. HRQoL was measured with the EQ-5D-3L and post-traumatic stress with the Impact of Event Scale-Revised (IES-R). Multinomial logistic regression identified factors independently associated with HRQoL deterioration. Concurrently, 36 semi-structured in-depth interviews were analysed using Braun and Clarke's thematic analysis framework.

**Results:**

Pain or discomfort was reported by 46.5% of participants (95% CI: 41.9–51.1%) and anxiety or depression by 36.9% (95% CI: 32.5–41.5%). On multivariable analysis, anxiety/depression was independently associated with larger household size (adjusted OR = 1.08 per person; 95% CI: 1.03–1.14; *p* = 0.002), greater distance to the DOTS clinic (adjusted OR = 1.03 per km; 95% CI: 1.01–1.05; *p* < 0.001), and appointment intervals of ≤ 14 days versus 15–30 days (adjusted OR = 2.86; 95% CI: 1.33–7.14; *p* = 0.011). Drug-resistant TB was strongly associated with activity limitation (adjusted OR = 27.7; 95% CI: 8.3–92.4). Qualitative analysis identified three themes: stigma intensification through symptom conflation with COVID-19; economic hardship obstructing treatment adherence and nutrition; and perceived inadequacy of government relief for chronically ill populations.

**Conclusions:**

In this cross-sectional study, HRQoL deterioration among urban TB patients in Bangladesh during the COVID-19 pandemic was significantly associated with modifiable structural factors. These preliminary findings suggest that appointment scheduling optimisation, service decentralisation, nutritional support, and psychosocial services may reduce pandemic-era HRQoL burden. Prospective longitudinal studies are needed to establish causality and inform programme design.

**Supplementary Information:**

The online version contains supplementary material available at 10.1007/s44197-026-00580-5.

## Introduction

Public health emergencies compound the vulnerability of people living with chronic conditions, exposing critical gaps in emergency management systems that are rarely designed to address co-occurring disease burdens. This study is directly relevant to United Nations Sustainable Development Goal (SDG) 3 (Good Health and Well-being), specifically Target 3.3, which calls for ending the tuberculosis (TB) epidemic by 2030, and Target 3.d, which requires strengthening national and global health security and emergency preparedness capacity [1]. Public health measures designed to contain pandemic transmission —including mobility restrictions and facility closures—can simultaneously disrupt care pathways for chronically ill populations, creating compounded health risks that general emergency protocols rarely address.

### Global and National Tuberculosis Burden

Tuberculosis remains the leading cause of mortality from a single infectious pathogen worldwide. According to the World Health Organization (WHO) Global Tuberculosis Report 2024, an estimated 10.8 million people fell ill with TB in 2023 and 1.25 million deaths were attributed to TB that year, including 161,000 among people living with HIV (PLHIV) [2]. The TB incidence rate increased by an estimated 4.6% between 2020 and 2023, reversing a decade of prior decline largely attributable to COVID-19-related disruptions to TB services. Bangladesh is among the eight countries that together account for more than two-thirds of the global TB burden [2].

In Bangladesh, the estimated TB incidence rate for all forms was 221 per 100,000 population in 2023—a figure unchanged since 2018—suggesting insufficient progress toward WHO End TB Strategy 2025 targets [3]. Approximately 379,000 incident TB cases were estimated in Bangladesh in 2022, with the National Tuberculosis Control Programme (NTP) reporting 301,000 notified cases, indicating a substantial detection gap [3, 4]. TB patients face clinically significant immunosuppression, side effects from prolonged drug regimens, catastrophic household costs, and pervasive social stigma—vulnerabilities substantially amplified during public health emergencies [5–9].

### COVID-19 Disruption to Tuberculosis Services

The COVID-19 pandemic created a dual public health crisis by simultaneously imposing acute pandemic risks on populations managing chronic disease while disrupting the health systems they depend upon. WHO estimated that COVID-19-related service disruptions could result in an additional 200,000 to 400,000 TB deaths globally through indirect effects [2]. In 2020, global TB case notifications fell by approximately 22% relative to pre-pandemic trends—a reduction reflecting diagnostic and service disruption rather than a genuine decline in incidence, as interrupted screening, laboratory closures, and patient reluctance to attend health facilities substantially reduced case-finding capacity [10].

Bangladesh experienced acute pandemic pressure during the study period. Dhaka accounted for approximately 40% of national COVID-19 cases during the first wave (March–September 2020) [3, 4]. Strict lockdowns, restrictions on public mobility, and economic contraction disproportionately affected informal economy workers—who constitute the majority of TB patients—by limiting income-generating activities, increasing transportation costs, and reducing access to treatment facilities and nutritious food [7, 8].

### Theoretical Framework: Syndemic Theory and the Triple Burden

Building on established syndemic theory, originally developed by Singer and Clair [11], which posits that two or more diseases or health conditions can interact synergistically within a specific population when contextual social forces such as poverty and structural disadvantage create conditions for their co-occurrence, this study applies a triple burden framework to TB patients during pandemic emergencies. Singer and Clair specified that for a syndemic interaction to occur, disease interactions must be cumulative, increasing the disease burden beyond what would be expected from non-interactive co-occurring conditions [4, 11].

The triple burden framework comprises: (1) the baseline clinical burden of active TB—immunosuppression, treatment side effects, and chronic disease management demands; (2) the acute pandemic burden—COVID-19 infection vulnerability, social distancing requirements, and disrupted services; and (3) poverty-driven resource constraints—overcrowded housing, transportation barriers, nutritional insecurity, and economic precarity. These three dimensions are conceptualised as mutually reinforcing rather than merely additive, consistent with syndemic theory [4, 11, 12]. This characterisation is consistent with the Social Determinants of Health framework, which situates individual health outcomes within structural conditions of inequality [13], and extends it by incorporating the dynamic interaction among concurrent health threats. The multiplicative interaction hypothesis remains a theoretical proposition requiring prospective testing with appropriate regression interaction terms; it is not confirmed by this cross-sectional study.

This framework is distinct from but complementary to Mendenhall's critical appraisal of syndemic theory [12], which calls for empirical data to test interaction claims. Our study contributes empirical evidence from a South Asian urban context where such data have been lacking.

### SDG Alignment

This study directly addresses SDG 3, Target 3.3 (ending TB by 2030) and Target 3.d (strengthening health security and emergency preparedness capacity) [1]. The lived experience of TB patients during COVID-19 illustrates critical gaps between SDG ambitions and implementation realities in low-income urban settings.

### Research Objectives and Gaps

Despite a growing body of literature documenting general population HRQoL deterioration during COVID-19, systematic evidence quantifying the specific and compounded vulnerability of TB patients in low-income settings—and identifying modifiable structural determinants—remains limited. This study addresses three objectives using a convergent mixed-methods approach [7]—applying the triple burden syndemic framework [4, 11] to TB-pandemic co-occurrence:

1. To measure the prevalence and patterns of HRQoL deterioration among TB patients during the COVID-19 first wave in Dhaka, Bangladesh.
2. To identify sociodemographic and clinical factors independently associated with HRQoL decline.
3. To examine, through qualitative inquiry, the lived mechanisms through which the pandemic affected TB patients' wellbeing and treatment engagement.

## Methods

### Study Design

A convergent parallel mixed-methods design was employed, as described by Creswell and Plano Clark [7]. The design comprised: (1) a cross-sectional quantitative component assessing HRQoL using validated instruments and identifying associated factors via multinomial logistic regression; and (2) a concurrent qualitative component examining pandemic-related experiences through semi-structured in-depth interviews. Both components were conducted simultaneously between August and September 2020. Findings were integrated at the interpretation stage to identify convergence, divergence, and complementarities—consistent with mixed-methods triangulation conventions. The study is reported in accordance with the Good Reporting of a Mixed Methods Study (GRAMMS) checklist [11].

### Study Setting

Dhaka is the capital city of Bangladesh and one of the most densely populated urban environments globally, with an estimated population of 21 million and a population density of approximately 44,500 persons per km^2^ [3, 14]. The city accounted for an estimated 40% of national COVID-19 cases during the study period [3, 4]. Dhaka is characterised by substantial informal economy employment, limited public health infrastructure relative to population size, and a large proportion of residents living in low-income neighbourhoods with overcrowded housing.

Twelve DOTS clinics—the standard TB treatment delivery platform in Bangladesh operated under the NTP—were selected across six municipal districts to ensure representation of socioeconomically diverse contexts, including slum settlements, middle-income neighbourhoods, and commercial districts [3, 15].

### Sampling and Sample Size

Consecutive sampling was employed for the quantitative component, enrolling all eligible patients presenting for scheduled treatment appointments at the 12 selected DOTS clinics during the study period. Consecutive sampling was selected for its feasibility and ethical appropriateness during an active pandemic, when probability-based recruitment was neither logistically viable nor ethically appropriate given infection exposure risks. This approach introduces a potential selection bias toward more resilient or healthcare-engaged patients, as individuals who were completely lost to follow-up or unable to access clinics—possibly due to more severe disease, greater distance, or poverty—are not represented in the sample. This limitation is discussed in Section 4.5.

Inclusion criteria were: age ≥ 18 years; confirmed TB diagnosis (any anatomical form); receipt of active treatment at recruitment; and capacity to provide informed written consent. Exclusion criteria were: severe cognitive impairment preventing interview participation; and hospitalisation at recruitment.

A total of 439 participants were enrolled. For a binary outcome with an expected prevalence of 37% and a minimum detectable odds ratio of 1.5, this sample achieves > 80% power at α = 0.05. Purposive sampling was used to select 36 participants for in-depth qualitative interviews, ensuring representation across sex, age group, TB type, HIV status, occupation, and residential distance. Thematic saturation—defined as the point at which no new codes or themes emerge—was monitored using a coding matrix tracking code frequency and co-occurrence across all transcripts. Saturation was reached by interview 33; interviews 34–36 generated no novel codes, confirming saturation consistent with guidelines for heterogeneous qualitative samples [11].

### Quantitative Data Collection

EQ-5D-3L: The EuroQol Five-Dimension Three-Level instrument (EQ-5D-3L) is a generic, validated HRQoL instrument used across more than 130 countries [16]. It measures five domains—mobility, self-care, usual activities, pain/discomfort, and anxiety/depression—each scored on a three-level ordinal scale (1 = no problems, 2 = some problems, 3 = extreme problems). A Bengali-validated version was administered [17]. Given that a Bangladesh-specific EQ-5D-3L value set was under development at the time of data collection and has only recently been formally disseminated (ARK Foundation, 2025), [18] health-state utility scores were derived using the Thai value set as a validated regional proxy—a common approach in South Asian settings [19]. Sensitivity analyses using the Japanese and UK value sets yielded consistent ordinal rankings of HRQoL domains.

IES-R: The Impact of Event Scale-Revised (IES-R) is a 22-item validated instrument measuring post-traumatic stress symptoms [10]. A Bengali-validated version was used. Scoring: total ≤ 32 = low stress; 33–38 = moderate; ≥ 39 = severe.

Supplementary sociodemographic and clinical variables collected included: age; sex; education; occupation; marital status; household size; distance to treatment centre (km, self-reported and verified with clinic address records); TB type (drug-sensitive [DS-TB] vs. drug-resistant [DR-TB]); anatomical localisation (pulmonary vs. extrapulmonary); HIV status; antiretroviral therapy (ART) receipt; treatment phase; and appointment interval category (≤ 14 days, 15–30 days, > 30 days).

Household size was used as a proxy for economic deprivation. In the Bangladesh urban context, large multi-person households in Dhaka typically reflect involuntary overcrowding driven by poverty rather than family preference, as evidenced by the inverse relationship between household size and per-capita food access documented in national household surveys [8, 14]. Direct income measurement was not collected given participants' reluctance to disclose income during the initial COVID-19 lockdown period.

Six trained research assistants conducted face-to-face interviews in Bengali at DOTS clinics following participants' routine clinical appointments. Each interview lasted 30–45 min. Data were recorded on tablet computers using REDCap electronic data capture. Quality assurance included daily data review, 10% re-interview for consistency verification, and weekly team meetings. All data collection followed institutional infection-control procedures, including surgical mask use, hand sanitisation, and physical distancing of ≥ 1 m.

### Qualitative Data Collection

A semi-structured interview guide was developed through iterative literature review and expert consultation with two public health physicians and one TB programme officer. Core domains included: perceptions of the pandemic; physical, psychological, social, and economic effects; transportation barriers; income disruption; changes in healthcare access; nutritional adequacy; stigma experiences; perceptions of government response; and coping strategies.

Interviews were conducted in Bengali by two experienced qualitative researchers. Sessions were audio-recorded with explicit written consent and lasted 25–40 min. Field notes recorded non-verbal contextual observations concurrently with audio recording; both data sources were triangulated during analysis. Audio recordings were transcribed verbatim in Bengali and translated to English by bilingual research team members. Back-translation of a 20% random sample was conducted by an independent bilingual researcher; discrepancies were resolved through team consensus. No significant semantic distortions were identified. All audio files and transcripts were stored on password-protected institutional servers accessible only to the named research team.

### Statistical Analysis

Descriptive statistics included means and standard deviations for continuous variables and frequencies with 95% confidence intervals for categorical variables. Normality was assessed via Shapiro–Wilk tests. Bivariate analyses used chi-square tests (or Fisher's exact test where cell counts < 5) to examine associations between EQ-5D-3L domain scores and sociodemographic/clinical variables.

Multinomial logistic regression was used to identify factors independently associated with HRQoL deterioration. For each EQ-5D-3L domain, responses were trichotomised (no problems vs. some problems vs. extreme problems) to retain clinically meaningful distinctions between severity levels. Multinomial regression was preferred over ordinal logistic regression because preliminary Brant tests indicated violation of the proportional odds assumption for several domains, making multinomial modelling more statistically appropriate despite the information-reduction trade-off. Independent variables entered a priori were: age, sex, education, occupation, household size, distance to treatment centre, TB type, HIV status, treatment phase, and appointment interval. Backward stepwise selection (*p* < 0 [20] retention threshold) was used; this approach is acknowledged to carry overfitting risk, and results are interpreted as hypothesis-generating. As a sensitivity analysis, all pre-specified variables were retained in a full model; the three primary associations (household size, clinic distance, appointment interval) remained consistent. Multicollinearity was assessed via Variance Inflation Factors (all VIF < 3.0). Adjusted odds ratios (aOR) with 95% confidence intervals are reported. Missing data were < 5% for all variables; complete case analysis was employed given that systematic bias from such low missingness is considered minimal, although a missing-at-random assumption is acknowledged and cannot be formally verified.

All analyses were conducted in R version 4.2.1 using packages MASS (multinomial regression), ggplot2 (visualisation), and psych (descriptive statistics). Two-tailed α = 0.05 was the significance threshold.

### Qualitative Analysis

Thematic analysis followed Braun and Clarke's six-phase framework: familiarisation; generating initial codes; searching for themes; reviewing themes; defining and naming themes; and producing the report.4 Two researchers independently coded all 36 transcripts using the RQDA package in R. An initial codebook was developed through open coding of the first eight interviews and subsequently applied and refined across all transcripts. A structured coding matrix tracked code frequency, co-occurrence, and participant characteristics, providing an explicit audit trail. Inter-coder reliability was assessed via Cohen's kappa (κ = 0.82, indicating substantial agreement). Both authors are Bangladeshi social scientists with prior experience in TB and poverty research; insider positionality facilitated rapport but also carries risk of confirmatory interpretation, mitigated through independent double-coding, reflexive journalling, and peer debriefing.

### Mixed-Methods Integration

Integration followed a convergent triangulation approach. Quantitative factor associations and qualitative themes were compared in a structured joint display (Table 4), examining: (a) convergence—qualitative data corroborating quantitative findings; (b) divergence—qualitative data challenging or complicating quantitative associations; and (c) complementarity—qualitative data adding mechanistic explanation to quantitative associations [7].

### Ethical Approval

Ethical approval was granted by the Jahangirnagar University Biosafety, Biosecurity and Ethical Clearance Committee (BBEC) [Protocol #BBEC, JU/M 2021/COVID-19/(8)1] and the icddr,b Ethics Review Committee (ERC) [Protocol #PR-21065]. All participants provided written informed consent. Participation was voluntary; participants received BDT 500 (~ USD 6) compensation for time. All identifying information was removed from datasets and transcripts. The study adhered to the Declaration of Helsinki and WHO Research Ethics Standards.

## Results

### Participant Characteristics

Of the 439 participants enrolled, 268 (61.0%) were male and 171 (39.0%) were female. The mean age was 35.1 years (SD: 11.8; range: 18–72). Most participants had completed primary or secondary education (65.0%) and were employed in the informal economy (40.1% informal/day labour; 20.0% garment/factory workers). Drug-sensitive TB (DS-TB) accounted for 90.0% of cases and pulmonary TB for 75.6%. The HIV-positive prevalence in this clinic-based sample was 24.0% (Table 1). Complete sociodemographic and clinical characteristics are presented in Table 1.

*Note on HIV prevalence: The 24% HIV prevalence substantially exceeds the national estimate of* <*0.1% for the general population. This reflects that DOTS clinics in Dhaka implement TB/HIV co-management under the NTP/NASP collaboration protocol, which mandates HIV testing for all TB patients and preferentially enrols known PLHIV. Findings on HIV status should be interpreted in this clinic-referral context and are not generalisable to all TB patients in Bangladesh* [4, 21]*.*

**Table 1:** Sociodemographic and clinical characteristics of study participants (*n* = 439)

Characteristic	*n*	% (95% CI)
Sex		
Male	268	61.0% (56.3–65.5%)
Female	171	39.0% (34.5–43.7%)
Age (years)		
Mean (SD)	35.1 (11.8)	—
Range	18–72	—
Education		
No formal education	89	20.3%
Primary (≤ Grade 5)	127	28.9%
Secondary (Grades 6–10)	158	36.0%
Higher secondary or above	65	14.8%
Occupation		
Informal/day labour	176	40.1%
Garment/factory worker	88	20.0%
Shopkeeper/trader	61	13.9%
Unemployed	58	13.2%
Student	31	7.1%
Other	25	5.7%
Household size: Mean (SD)	6.1 (2.4)	Range: 1–15
Distance to DOTS clinic (km): Mean (SD)	6.96 (8.3)	Range: 2–100
TB type		
Drug-sensitive TB (DS-TB)	395	90.0%
Drug-resistant TB (DR-TB)	44	10.0%
Anatomical localisation		
Pulmonary	332	75.6%
Extrapulmonary	107	24.4%
HIV status		
HIV-positive	105	24.0%
HIV-negative	334	76.0%
ART receipt (HIV-positive only)	95/105	90.5%
Treatment phase		
Intensive phase	198	45.1%
Continuation phase	241	54.9%
Appointment interval		
≤ 14 days	139	31.7%
15–30 days	221	50.3%
> 30 days	79	18.0%

### HRQoL Deterioration: EQ-5D-3L Domain Findings

Pain or discomfort was the most prevalent domain of HRQoL impairment, affecting 46.5% of participants (95% CI: 41.9–51.1%), followed by anxiety or depression (36.9%; 95% CI: 32.5–41.5%). Functional domains—mobility, usual activities, and self-care—showed substantially lower rates of impairment. Full domain distributions are presented in Table 2.

**Table 2:** EQ-5D-3L Domain Score Distribution (*n* = 439)

EQ-5D-3L Domain	No Problems *n* (%)	Some Problems *n* (%)	Extreme Problems *n* (%)	Any Problems % (95% CI)
Pain/discomfort	235 (53.5%)	137 (31.2%)	67 (15.3%)	46.5% (41.9–51.1%)
Anxiety/depression	277 (63.1%)	145 (33.1%)	17 (3.8%)	36.9% (32.5–41.5%)
Mobility	348 (79.3%)	72 (16.4%)	19 (4.3%)	20.7% (17.0–24.9%)
Usual activities	359 (81.8%)	61 (13.9%)	19 (4.3%)	18.2% (14.7–22.3%)
Self-care	399 (90.9%)	31 (7.1%)	9 (2.0%)	9.1% (6.7–12.2%)

Post-traumatic stress symptom burden: 27.3% of participants scored ≥ 33 on the IES-R (moderate-to-severe), indicating significant post-traumatic stress symptom load even during the post-lockdown relaxation phase of August–September 2020.

### Multivariable Factors Associated with HRQoL Deterioration

Three sociodemographic factors were independently associated with anxiety/depression on multivariable analysis, and two clinical factors were associated with activity limitation (Table 3). Variables not retained in the final models (all *p* > 0.05 after backward selection) included education level, sex, marital status, and TB anatomical localisation.

**Table 3:** Multinomial logistic regression — factors associated with anxiety/depression and activity limitation

Variable	Outcome	Adjusted OR	95% CI	*p*-value
Household size (per person)	Anxiety/depression vs. none	1.08	1.03–1.14	0.002
Distance to DOTS clinic (per km)	Anxiety/depression vs. none	1.03	1.01–1.05	< 0.001
Appointment ≤ 14 days (ref: 15–30 days)	Anxiety/depression vs. none	2.86	1.33–7.14	0.011
Drug-resistant TB (ref: DS-TB)	Activity limitation vs. none	27.7	8.3–92.4	< 0.001
Age > 59 years (ref: 18–39 years)	Activity limitation vs. none	5.40	1.5–19.8	0.011
HIV-positive (ref: HIV-negative)	Anxiety/depression vs. none	0.45	0.22–0.92	0.029

Variables not retained in final models (*p* > 0.05): education, sex, marital status, TB anatomical localization

Household size: Each additional household member was associated with an 8% increase in the odds of anxiety/depression (aOR = 1.08; 95% CI: 1.03–1.14; *p* = 0.002). In the Bangladesh urban context, larger household size is an established proxy for poverty-driven overcrowding; however, direct income or per-room density measurement was not available, and this interpretation should be regarded as inferential [8, 14].

Distance to DOTS clinic: Each additional kilometre from the treatment clinic was associated with a 3% increase in the odds of anxiety/depression (aOR = 1.03; 95% CI: 1.01–1.05; *p* < 0.001). This effect size exceeds the 1–1.5% per km reported in non-pandemic-era TB studies from South Asia and sub-Saharan Africa [1, 9], consistent with pandemic-specific transportation disruption amplifying a pre-existing barrier.

Appointment interval: Patients with appointment intervals of ≤ 14 days showed approximately 2.9-fold higher odds of anxiety/depression compared with those on 15–30 day intervals (aOR = 2.86; 95% CI: 1.33–7.14; *p* = 0.011). This association requires cautious interpretation: the cross-sectional design cannot exclude reverse causality—clinicians may assign more frequent appointments to patients with greater clinical complexity or psychological instability. This is best characterised as an optimisation finding—a hypothesis that, for clinically stable patients, more frequent visit schedules may impose transportation and economic burdens contributing to anxiety, warranting prospective investigation [22, 23].

DR-TB and activity limitation: DR-TB patients showed 27.7-fold greater odds of activity limitation compared with DS-TB patients (aOR = 27.7; 95% CI: 8.3–92.4; *p* < 0.001), reflecting the compounding effect of more intensive treatment regimens [9].

HIV/TB co-infection: HIV-positive patients showed 55% lower odds of anxiety/depression (aOR = 0.45; 95% CI: 0.22–0.92; *p* = 0.029). This counter-intuitive association may reflect non-exclusive mechanisms including prior chronic illness adaptation, structured ART adherence counselling providing psychosocial support, and clinic-based selection effects. These interpretations are speculative and require prospective investigation.

### Qualitative Findings: Mechanisms of the Triple Burden

Three major themes and six sub-themes emerged from thematic analysis of 36 in-depth interviews.

Theme 1 — Stigma Intensification through Symptom Conflation: TB's characteristic symptoms (persistent cough, fever, night sweats) overlap substantially with early COVID-19 presentations. This similarity triggered renewed and intensified stigmatisation of TB patients by community members, employers, and family members. A 34-year-old male rickshaw puller stated: "Once you cough, people move away immediately. They think you have COVID-19. TB has been forgotten; all attention is on coronavirus." A 41-year-old female garment worker reported: "My husband doesn't allow me near him because of the cough. He says I might give him corona. We eat separately, sleep separately. I feel like a stranger in my own home." This domestic exclusion compounded treatment adherence difficulties by reducing household support for health-seeking behavior [7, 23]. Sub-themes: (1a) public space exclusion; (1b) domestic exclusion.

Theme 2 — Economic Hardship as a Structural Barrier to Treatment and Nutrition: Pandemic-era lockdowns disproportionately disrupted the livelihoods of informal economy workers (76% of the sample). A 38-year-old street vendor stated: "My shop was closed for three months. No income, no savings. How can I go to the hospital when I cannot feed my children?" Transportation costs emerged as a critical proximate barrier. A 29-year-old artist explained: "No events now, no work. Finding transport to the centre is a major problem because transport is so expensive now. Sometimes we don't come on scheduled dates because we lack money for transportation." A 45-year-old day labourer added: "I cannot eat foods my doctor recommends —milk, eggs, fruits. I eat only rice and lentils." This directly corroborates the quantitative finding linking greater clinic distance to worse HRQoL outcomes [6]. Sub-themes: (2a) income loss and food insecurity; (2b) transportation unaffordability.

Theme 3 — Perceived Inadequacy of Government Emergency Response for Chronically Ill Populations: Government pandemic mitigation measures were broadly perceived as insufficient for TB patients whose needs differed structurally from those of the general population. A 52-year-old shopkeeper observed: "These lockdowns are for wealthy people who have savings. We eat day to day. When you close everything, we starve." A 37-year-old patient stated: "The state should help its population, especially TB patients who are rejected by bosses for fear of infection. We need specific support—medicine delivery, food, transport vouchers." Triangulation with NTP reports and DGHS COVID-19 documentation confirms that Bangladesh's 2020 emergency response packages did not include TB-specific provisions for treatment continuity, nutritional support, or transport subsidy [3, 8, 14, 15]. Sub-themes: (3a) perception of policy equity failure; (3b) calls for disease-specific targeted relief.

### Mixed-Methods Integration: Joint Display

Table 4 presents the structured joint display of quantitative and qualitative findings by triple burden dimension, identifying patterns of convergence, complementarity, and divergence.

**Table 4:** Joint display of quantitative and qualitative findings by triple burden dimension

Triple Burden Dimension	Quantitative Finding	Qualitative Corroboration	Integration Interpretation
Poverty-driven crowding	HH size aOR 1.08/person (*p* = 0.002)	Theme 2: economic collapse, shared housing	Convergence: economic mechanisms corroborated
Acute pandemic constraint	Distance aOR 1.03/km (*p* < 0.001)	Theme 2: transport unaffordability	Convergence: same barrier identified by both methods
Appointment burden	≤ 14-day interval aOR 2.86 vs. 15–30 days	Theme 2: full-day clinic trips impose cost/time burden	Convergence with caveat: reverse causality cannot be excluded
Stigma	Not directly measured	Theme 1: dual stigma from symptom conflation	Complementarity: qualitative adds dimension not captured by EQ-5D-3L
Government response	Not tested quantitatively	Theme 3: relief inadequacy for chronically ill	Complementarity: contextual finding confirmed by policy triangulation
Clinical severity (DR-TB)	DR-TB aOR 27.7 for activity limitation	Not prominent in qualitative data	Divergence: clinical severity dominates quantitatively

## Discussion

### Situating the Findings Within Syndemic Theory

This study contributes empirical evidence for the applicability of syndemic theory—specifically Singer and Clair's framework of interacting health conditions amplified by structural disadvantage [4, 11]—to TB-pandemic co-occurrence in a South Asian urban setting. The convergent findings (quantitative associations corroborated by qualitative mechanisms) are consistent with, rather than establishing, a syndemic interaction: poverty proxies, service access barriers, and stigma contributed independently to HRQoL deterioration in ways that mutually reinforce each other across the three burden dimensions. However, formal testing of multiplicative interaction requires prospective data with appropriate regression interaction terms, as called for by Mendenhall's critical appraisal of syndemic research [12].

The triple burden framework is distinct from, but complementary to, the Social Determinants of Health (SDH) framework [13], which identifies structural conditions shaping health outcomes but does not model dynamic disease-disease interaction, and the Andersen Behavioural Model, which emphasises predisposing, enabling, and need factors without explicitly modelling acute-chronic health threat interaction. The triple burden framework integrates these perspectives for the specific context of chronic disease patients during public health emergencies.

### Appointment Interval: an Optimisation Finding

The observed association between appointment intervals of ≤ 14 days and worse anxiety outcomes (aOR = 2.86) represents an optimisation finding rather than a causal effect. The cross-sectional design cannot establish causal direction: clinicians may assign more frequent appointments to patients with greater clinical complexity or psychological instability (reverse causality), which would produce the observed association without any mechanism through which visit frequency itself causes harm. The more appropriate policy-relevant interpretation is: for clinically stable TB patients during pandemic conditions, contact intervals balancing monitoring needs against transportation and economic burden may warrant evaluation as a mechanism to reduce HRQoL impairment, without compromising adherence.

This is broadly consistent with evidence from TB programmes in Cambodia, Rwanda, and Uganda demonstrating that extended medication dispensing intervals of 30 days achieve equivalent treatment success rates with improved patient satisfaction [22, 23]. However, those studies were not conducted during pandemic conditions and involved different outcome measures; direct comparability is limited. A prospective randomised study of appointment interval effects on both HRQoL and treatment outcomes is needed before clinical recommendations can be made.

### Comparison With Prior Literature

The observed prevalence of pain/discomfort (46.5%) and anxiety/depression (36.9%) is at the upper range of estimates from non-pandemic-era TB studies from South Asia and sub-Saharan Africa, where anxiety/depression prevalence ranges from 25–40% [5, 6, 13]. This is consistent with the hypothesis that pandemic conditions contributed additional burden beyond the baseline disease-related HRQoL impairment. COVID-19 survivor studies in Bangladesh have similarly documented lower quality of life scores in psychological domains, with greater impairment among individuals with chronic conditions [3, 24].

The distance-anxiety association effect size (3% per km) exceeds the 1–1.5% per km reported in prior non-pandemic settings [1, 9], consistent with pandemic-specific transportation disruption amplifying a pre-existing barrier. Bangladesh's extreme urban density (44,500 persons/km^2^), dependence on informal economy livelihoods (> 76% of this sample), and high TB burden collectively amplify the mechanisms identified here relative to settings with more formal social protection systems [3, 7, 8].

### Evidence-Informed Intervention Directions

The following are offered as evidence-informed hypotheses for prospective evaluation, not definitive guidelines, given this study's cross-sectional, single-city design [3, 15].

#### **Service Decentralisation**

Expanding DOTS coverage through nurse-led satellite clinics or community health worker-based medication delivery may address the distance-anxiety association, consistent with NTP's National Strategic Plan 2024–2030 provisions for community-based TB care [15]. Feasibility modelling—including resource mapping and transport infrastructure analysis—would be required before operational implementation.

#### **Appointment Interval Optimisation**

Evidence-based stability criteria to identify patients for whom extended dispensing intervals (21–30 days) may be clinically appropriate should be developed and tested in prospective trials measuring both HRQoL and treatment outcomes. Telemedicine-supported follow-up between in-person visits could maintain monitoring while reducing visit burden.

#### **Nutritional Support Integration**

Given nutritional insecurity's prominence in qualitative data and the established impact of malnutrition on TB treatment efficacy [5, 6], expansion of nutritional support to all active TB patients (not only DR-TB patients as per current NTP policy) warrants evaluation.

#### **Psychosocial Services**

The 36.9% anxiety/depression prevalence and 27.3% moderate-to-severe IES-R rate indicate high demand for psychological support. Integration of lay counsellors and stigma-reduction communication campaigns into DOTS clinic operations should be piloted and evaluated.

#### **TB-Specific Emergency Provisions**

Future pandemic preparedness plans should incorporate TB-specific provisions for treatment continuity, medication home delivery, transport vouchers, and income support. Generic emergency relief was perceived as structurally insufficient for chronically ill populations in this study and corroborated by policy documentation showing the absence of TB-specific measures in Bangladesh's 2020 emergency response packages [25].

### Limitations

The following limitations should be considered when interpreting these findings:

4. Cross-sectional design: Causal inference is not possible. The direction and nature of associations between structural factors and HRQoL cannot be established from these data alone.
5. Selection bias: Consecutive clinic sampling excluded patients lost to follow-up, hospitalised, or unable to attend—potentially the most vulnerable subgroup. The sample likely overrepresents more resilient or healthcare-engaged patients.
6. Single urban setting: Findings from Dhaka cannot be directly generalised to rural Bangladeshi settings or other countries.
7. Self-reported measures and recall bias: EQ-5D-3L scores, appointment frequency, and distance data were self-reported and subject to social desirability and recall biases.
8. Unmeasured confounders: Income level, employment status, per-room density, adherence behaviour, and provider practice changes were not directly measured. Residual confounding cannot be excluded.
9. Reverse causality: The appointment frequency association is subject to reverse causality and should not be interpreted as a causal finding.
10. HIV prevalence: The 24% HIV prevalence reflects clinic-based enrichment and does not represent community TB patients.
11. Temporal specificity: Data were collected during the COVID-19 first wave only. Subsequent waves, vaccination, and system adaptation may have altered the patterns observed.
12. Qualitative transferability: Thematic transferability to other settings should be applied cautiously.

### Future Research Directions

Priority research directions include: (1) longitudinal cohort studies tracking HRQoL trajectories across pandemic phases; (2) prospective randomised trials of appointment interval optimisation and service decentralisation with dual HRQoL and treatment outcome endpoints; (3) economic evaluation of integrated support packages; (4) extension to rural Bangladeshi settings and other high-burden low- and middle-income countries; (5) comparative study across chronic diseases; and (6) formal syndemic interaction modelling using product terms in regression analyses.

## Conclusions

This convergent mixed-methods study of 439 TB patients attending DOTS clinics in Dhaka, Bangladesh, during the COVID-19 first wave found that 46.5% reported pain or discomfort and 36.9% reported anxiety or depression. Three modifiable structural factors were independently associated with anxiety/depression on multivariable analysis: larger household size (a proxy for poverty-driven crowding), greater distance to DOTS clinics (amplified by pandemic transportation disruption), and appointment intervals of ≤ 14 days compared with 15–30 days (an association that may reflect clinical selection or visit burden, though causality cannot be established cross-sectionally). DR-TB was strongly associated with activity limitation.

Qualitative findings—intensified stigma through symptom conflation with COVID-19, economic hardship obstructing treatment adherence and nutrition, and perceived inadequacy of general emergency relief for chronically ill populations—provided mechanistic context for the quantitative associations and identified dimensions of vulnerability not captured by HRQoL instruments alone. Triangulation with NTP policy documentation confirmed that Bangladesh's 2020 pandemic emergency response did not include TB-specific provisions.

These preliminary, cross-sectional findings, situated within a syndemic framework applying established theory to TB-pandemic co-occurrence in a South Asian urban context, offer exploratory evidence to inform the development of future emergency preparedness guidelines for TB programmes. Specific provisions—treatment continuity, optimised appointment scheduling for stable patients, nutritional support, psychosocial services, and transport assistance—should be evaluated through prospective studies before operational implementation. Causal and generalisable conclusions require longitudinal designs, multi-site validation, and formal syndemic interaction modelling. Key limitations include consecutive clinic-based sampling, the cross-sectional design precluding causal inference, single urban setting, self-reported HRQoL measures subject to recall bias, and unmeasured confounders.

## Supplementary Information

Below is the link to the electronic supplementary material.

Supplementary file1 (DOCX 243 KB)

## Data Availability

The de-identified quantitative dataset and anonymized qualitative transcripts generated during this study are available from the corresponding author upon reasonable request, subject to formal data-sharing agreement and compliance with Bangladesh Medical Research Council ethical guidelines. Access requests must include justification of proposed data use and institutional affiliation. Data will be provided in fully de-identified format to ensure participant confidentiality and protection. All requests will be processed within 4–6 weeks of submission. Raw data with identifiable information will be securely retained for seven years post-publication, and then destroyed per involved institutional policy.
