# Supplementary material for: Health-Related Quality of Life Deterioration Among Tuberculosis Patients During the COVID-19 Pandemic: a Convergent Mixed-Methods Study of Syndemic Vulnerability in Urban Bangladesh
Source: J Epidemiol Glob Health. 2026 May 25;16(1):104. doi: 10.1007/s44197-026-00580-5 (PMC13478039; doi:10.1007/s44197-026-00580-5)
Supplement: Supplementary file 1 [file 44197_2026_580_MOESM1_ESM.docx]

**Supplementary File 1: Complete Study Questionnaire**

**Study Title:** Dual-disease emergency management during pandemic crisis: Health-related quality of life deterioration patterns and evidence-based interventions for vulnerable tuberculosis populations under COVID-19 stress in Bangladesh

**Principal Investigator:** Santu Das, Department of Economics, Jahangirnagar University

**Study Period:** August – September 2020

**Ethics Approval:**

- Jahangirnagar University BBEC Protocol #BBEC, JU/M 2021/COVID-19/(8)1
- icddr,b Ethics Review Committee Protocol #PR-21065

**Instructions for Data Collectors**

**Interview Duration:** 30-45 minutes

**Language:** Bengali (this is the English translation)

**Setting:** Face-to-face interview at TB Diagnosis and Treatment Centers (DTCs) following routine clinical appointments

**Data Recording:** REDCap electronic data capture system on tablet computers

**Participant Requirements:**

- Age ≥18 years
- Diagnosed tuberculosis (any form)
- Currently receiving active treatment
- Able to provide informed consent
- Not hospitalized at time of recruitment
- No severe cognitive impairment

**Informed Consent:** Must be obtained before beginning the questionnaire

**Compensation:** 500 Bangladeshi Taka (~$6 USD) provided after interview completion

**SECTION A: PARTICIPANT IDENTIFICATION AND RECRUITMENT**

**A1. Participant ID Number:** |***|***|***|***|___|

**A2. Interview Date:** Day |***|***| Month |***|***| Year |***|***|***|***|

**A3. Interview Start Time:** |***|***|:|***|***|

**A4. Interview End Time:** |***|***|:|***|***|

**A5. Data Collector Name:** ___________________________

**A6. Data Collector ID:** |***|***|___|

**A7. Treatment Center Name:**

1. ☐ DTC 1 – Mohammadpur
2. ☐ DTC 2 – Mirpur
3. ☐ DTC 3 – Uttara
4. ☐ DTC 4 – Dhanmondi
5. ☐ DTC 5 – Gulshan
6. ☐ DTC 6 – Motijheel
7. ☐ DTC 7 – Old Dhaka
8. ☐ DTC 8 – Tejgaon
9. ☐ DTC 9 – Khilgaon
10. ☐ DTC 10 – Rampura
11. ☐ DTC 11 – Savar
12. ☐ DTC 12 – Kamrangirchar

**A8. District:** ___________________________

**SECTION B: SOCIODEMOGRAPHIC CHARACTERISTICS**

**B1. What is your current age?**

Age in years: |***|***| years

**B2. What is your sex?**

1. ☐ Male
2. ☐ Female
3. ☐ Other (specify): _______________

**B3. What is your highest level of education completed?**

1. ☐ No formal education
2. ☐ Primary (Class 1-5)
3. ☐ Secondary (Class 6-10)
4. ☐ Higher Secondary (Class 11-12)
5. ☐ Bachelor's degree
6. ☐ Master's degree or higher
7. ☐ Religious education only
8. ☐ Other (specify): _______________

**B4. What is your current marital status?**

1. ☐ Never married
2. ☐ Currently married
3. ☐ Separated
4. ☐ Divorced
5. ☐ Widowed
6. ☐ Living with partner (not married)

**B5. What is your primary occupation?**

1. ☐ Unemployed
2. ☐ Student
3. ☐ Homemaker
4. ☐ Rickshaw/auto-rickshaw puller
5. ☐ Garment worker
6. ☐ Day laborer
7. ☐ Shopkeeper/street vendor
8. ☐ Factory worker
9. ☐ Office worker/clerk
10. ☐ Professional (doctor, teacher, engineer)
11. ☐ Skilled tradesperson (carpenter, electrician)
12. ☐ Driver (taxi, truck, bus)
13. ☐ Security guard
14. ☐ Domestic worker
15. ☐ Farmer
16. ☐ Artist/performer
17. ☐ Self-employed business
18. ☐ Retired
19. ☐ Unable to work due to illness
20. ☐ Other (specify): _______________

**B6. How many people currently live in your household, including yourself?**

Number of household members: |***|***| persons

**B7. How many rooms does your household have for sleeping?**

Number of sleeping rooms: |***|***| rooms

*(Interviewer calculate: Persons per room = B6 ÷ B7)*

**B8. What is the approximate distance from your home to this TB treatment center?**

Distance: |***|***|___| kilometers

*(If less than 1 km, write 001)*

**B9. How do you usually travel to this TB treatment center?**

1. ☐ Walking only
2. ☐ Bicycle
3. ☐ Rickshaw
4. ☐ Auto-rickshaw/CNG
5. ☐ Bus
6. ☐ Train
7. ☐ Private car/motorcycle
8. ☐ Combination of multiple transport (specify): _______________

**B10. How much does it typically cost you for a one-way trip to this treatment center?**

Cost: |***|***|___| Taka

*(If walking or free, write 000)*

**B11. Approximately how long does it take you to travel from home to this treatment center?**

Travel time: |***|***| hours |***|***| minutes

**B12. What type of area do you live in?**

1. ☐ Urban/city center
2. ☐ Semi-urban/suburban
3. ☐ Rural village
4. ☐ Slum settlement
5. ☐ Other (specify): _______________

**B13. What is your approximate total monthly household income from all sources?**

1. ☐ Less than 5,000 Taka
2. ☐ 5,000 – 10,000 Taka
3. ☐ 10,001 – 15,000 Taka
4. ☐ 15,001 – 20,000 Taka
5. ☐ 20,001 – 25,000 Taka
6. ☐ 25,001 – 30,000 Taka
7. ☐ 30,001 – 40,000 Taka
8. ☐ 40,001 – 50,000 Taka
9. ☐ More than 50,000 Taka
10. ☐ Don't know/Prefer not to answer

**SECTION C: TUBERCULOSIS CLINICAL CHARACTERISTICS**

**C1. When were you first diagnosed with tuberculosis?**

Date of diagnosis: Day |***|***| Month |***|***| Year |***|***|***|***|

*(If exact date unknown, record month and year only)*

**C2. How long have you been on TB treatment (in months)?**

Duration: |***|***| months

**C3. What type of tuberculosis do you have?**

1. ☐ Drug-sensitive TB (first-line treatment)
2. ☐ Drug-resistant TB (MDR-TB or XDR-TB)
3. ☐ Don't know
4. ☐ Other (specify): _______________

**C4. What is the anatomical location of your tuberculosis?**

1. ☐ Pulmonary TB (lungs only)
2. ☐ Extra-pulmonary TB (specify site): _______________
3. ☐ Both pulmonary and extra-pulmonary
4. ☐ Don't know

**C5. Is this your first time being treated for tuberculosis?**

1. ☐ Yes, first time (skip to C7)
2. ☐ No, I have been treated before

**C6. If you have been treated before, how many times?**

Number of previous TB treatment episodes: |***|***| times

**C7. How was your tuberculosis diagnosed? (Multiple answers possible)**

1. ☐ Sputum smear microscopy
2. ☐ Sputum culture
3. ☐ GeneXpert MTB/RIF test
4. ☐ Chest X-ray
5. ☐ Clinical symptoms only
6. ☐ Other test (specify): _______________
7. ☐ Don't know

**C8. What is your current treatment phase?**

1. ☐ Intensive phase (first 2-3 months)
2. ☐ Continuation phase (after intensive phase)
3. ☐ Don't know

**C9. What is your current TB treatment regimen? (Record medication names if known)**

Medications: _________________________________________________

**C10. How often do you currently have scheduled clinic appointments for TB treatment?**

1. ☐ Every week (7 days or less)
2. ☐ Every 8-14 days
3. ☐ Every 15-21 days
4. ☐ Every 22-30 days
5. ☐ More than 30 days
6. ☐ Irregular/as needed
7. ☐ Other (specify): _______________

**C11. Have you missed any scheduled TB clinic appointments in the past 3 months?**

1. ☐ No, attended all appointments
2. ☐ Yes, missed 1-2 appointments
3. ☐ Yes, missed 3-5 appointments
4. ☐ Yes, missed more than 5 appointments

**C12. If yes, what were the main reasons for missing appointments? (Multiple answers possible)**

1. ☐ Not applicable (did not miss appointments)
2. ☐ Transportation not available
3. ☐ Could not afford transportation cost
4. ☐ Lockdown/movement restrictions
5. ☐ Fear of COVID-19 infection
6. ☐ Feeling too sick to travel
7. ☐ Work or family obligations
8. ☐ Forgot appointment date
9. ☐ Treatment center closed or not accessible
10. ☐ Other (specify): _______________

**C13. Are you currently taking all your TB medications as prescribed?**

1. ☐ Yes, taking all medications regularly
2. ☐ Sometimes miss doses
3. ☐ Frequently miss doses
4. ☐ Have stopped taking medications

**C14. If you are missing doses, what are the main reasons? (Multiple answers possible)**

1. ☐ Not applicable (taking medications regularly)
2. ☐ Side effects from medications
3. ☐ Too many pills to take
4. ☐ Ran out of medications
5. ☐ Forgot to take them
6. ☐ Feeling better, think I don't need them
7. ☐ Cannot afford to buy food to take with medications
8. ☐ Other (specify): _______________

**C15. Have you experienced any side effects from TB medications?**

1. ☐ No side effects
2. ☐ Yes (specify): _______________________________________

Common side effects to ask about:

- ☐ Nausea/vomiting
- ☐ Loss of appetite
- ☐ Joint pain
- ☐ Numbness/tingling in hands or feet
- ☐ Vision problems
- ☐ Hearing problems
- ☐ Skin rash
- ☐ Jaundice (yellowing of skin/eyes)
- ☐ Other: _______________

**C16. Have you been tested for HIV/AIDS?**

1. ☐ Yes, tested HIV-positive
2. ☐ Yes, tested HIV-negative
3. ☐ No, never tested
4. ☐ Don't know/Prefer not to answer

**C17. If HIV-positive, are you currently receiving antiretroviral therapy (ART)?**

1. ☐ Not applicable (HIV-negative or not tested)
2. ☐ Yes, currently on ART
3. ☐ No, not on ART
4. ☐ Don't know

**C18. Do you have any other chronic health conditions? (Multiple answers possible)**

1. ☐ No other conditions
2. ☐ Diabetes
3. ☐ Hypertension
4. ☐ Heart disease
5. ☐ Lung disease (asthma, COPD)
6. ☐ Kidney disease
7. ☐ Liver disease
8. ☐ Cancer
9. ☐ Mental health condition
10. ☐ Other (specify): _______________

**SECTION D: COVID-19 PANDEMIC IMPACT**

**D1. During the COVID-19 pandemic, have you or anyone in your household been infected with COVID-19?**

1. ☐ No, no one in household
2. ☐ Yes, I was infected
3. ☐ Yes, household member(s) infected (not me)
4. ☐ Yes, both I and household member(s) infected
5. ☐ Don't know

**D2. Have you been tested for COVID-19?**

1. ☐ Yes, tested positive
2. ☐ Yes, tested negative
3. ☐ No, never tested
4. ☐ Don't know

**D3. How worried are you about getting COVID-19?**

1. ☐ Not at all worried
2. ☐ Slightly worried
3. ☐ Moderately worried
4. ☐ Very worried
5. ☐ Extremely worried

**D4. Has the COVID-19 pandemic affected your ability to access TB treatment?**

1. ☐ No effect
2. ☐ Yes, minor difficulties
3. ☐ Yes, major difficulties
4. ☐ Yes, completely unable to access at times

**D5. Has the COVID-19 pandemic affected your household income?**

1. ☐ No change
2. ☐ Slightly decreased (less than 25%)
3. ☐ Moderately decreased (25-50%)
4. ☐ Severely decreased (50-75%)
5. ☐ Almost completely lost (more than 75%)
6. ☐ Increased

**D6. Have you or your household received any government assistance during the COVID-19 pandemic?**

1. ☐ Yes, received assistance (specify type): _______________
2. ☐ No, did not receive assistance
3. ☐ Applied but did not receive
4. ☐ Did not apply

**D7. Has the COVID-19 pandemic affected your ability to afford adequate food?**

1. ☐ No change in food access
2. ☐ Sometimes cannot afford adequate food
3. ☐ Often cannot afford adequate food
4. ☐ Rarely have adequate food

**SECTION E: HEALTH-RELATED QUALITY OF LIFE (EQ-5D-3L)**

**Instructions:** Under each heading, please tick the ONE box that best describes your health TODAY.

**E1. MOBILITY**

1. ☐ I have no problems in walking about
2. ☐ I have some problems in walking about
3. ☐ I am confined to bed

**E2. SELF-CARE**

1. ☐ I have no problems with self-care
2. ☐ I have some problems washing or dressing myself
3. ☐ I am unable to wash or dress myself

**E3. USUAL ACTIVITIES (e.g. work, study, housework, family or leisure activities)**

1. ☐ I have no problems with performing my usual activities
2. ☐ I have some problems with performing my usual activities
3. ☐ I am unable to perform my usual activities

**E4. PAIN/DISCOMFORT**

1. ☐ I have no pain or discomfort
2. ☐ I have moderate pain or discomfort
3. ☐ I have extreme pain or discomfort

**E5. ANXIETY/DEPRESSION**

1. ☐ I am not anxious or depressed
2. ☐ I am moderately anxious or depressed
3. ☐ I am extremely anxious or depressed

**E6. EQ VISUAL ANALOGUE SCALE (EQ-VAS)**

We would like to know how good or bad your health is TODAY.

This scale is numbered from 0 to 100.

- **100** means the **best health you can imagine**
- **0** means the **worst health you can imagine**

Please mark an X on the scale to indicate how your health is TODAY.

**YOUR HEALTH TODAY =** |***|***|___| (0-100)

**SECTION F: IMPACT OF EVENT SCALE - REVISED (IES-R)**

**Instructions:** Below is a list of difficulties people sometimes have after stressful life events. Please read each item, and then indicate how distressing each difficulty has been for you DURING THE PAST SEVEN DAYS with respect to the COVID-19 pandemic and your experience as a tuberculosis patient. How much were you distressed or bothered by these difficulties?

**Response Scale:**

- 0 = Not at all
- 1 = A little bit
- 2 = Moderately
- 3 = Quite a bit
- 4 = Extremely

| Item | Question | 0 | 1 | 2 | 3 | 4 |
| --- | --- | --- | --- | --- | --- | --- |
| F1 | Any reminder brought back feelings about it | ☐ | ☐ | ☐ | ☐ | ☐ |
| F2 | I had trouble staying asleep | ☐ | ☐ | ☐ | ☐ | ☐ |
| F3 | Other things kept making me think about it | ☐ | ☐ | ☐ | ☐ | ☐ |
| F4 | I felt irritable and angry | ☐ | ☐ | ☐ | ☐ | ☐ |
| F5 | I avoided letting myself get upset when I thought about it or was reminded of it | ☐ | ☐ | ☐ | ☐ | ☐ |
| F6 | I thought about it when I didn't mean to | ☐ | ☐ | ☐ | ☐ | ☐ |
| F7 | I felt as if it hadn't happened or wasn't real | ☐ | ☐ | ☐ | ☐ | ☐ |
| F8 | I stayed away from reminders about it | ☐ | ☐ | ☐ | ☐ | ☐ |
| F9 | Pictures about it popped into my mind | ☐ | ☐ | ☐ | ☐ | ☐ |
| F10 | I was jumpy and easily startled | ☐ | ☐ | ☐ | ☐ | ☐ |
| F11 | I tried not to think about it | ☐ | ☐ | ☐ | ☐ | ☐ |
| F12 | I was aware that I still had a lot of feelings about it, but I didn't deal with them | ☐ | ☐ | ☐ | ☐ | ☐ |
| F13 | My feelings about it were kind of numb | ☐ | ☐ | ☐ | ☐ | ☐ |
| F14 | I found myself acting or feeling like I was back at that time | ☐ | ☐ | ☐ | ☐ | ☐ |
| F15 | I had trouble falling asleep | ☐ | ☐ | ☐ | ☐ | ☐ |
| F16 | I had waves of strong feelings about it | ☐ | ☐ | ☐ | ☐ | ☐ |
| F17 | I tried to remove it from my memory | ☐ | ☐ | ☐ | ☐ | ☐ |
| F18 | I had trouble concentrating | ☐ | ☐ | ☐ | ☐ | ☐ |
| F19 | Reminders of it caused me to have physical reactions, such as sweating, trouble breathing, nausea, or a pounding heart | ☐ | ☐ | ☐ | ☐ | ☐ |
| F20 | I had dreams about it | ☐ | ☐ | ☐ | ☐ | ☐ |
| F21 | I felt watchful and on-guard | ☐ | ☐ | ☐ | ☐ | ☐ |
| F22 | I tried not to talk about it | ☐ | ☐ | ☐ | ☐ | ☐ |

**IES-R Scoring (for data analyst):**

**Intrusion Subscale** (items F1, F3, F6, F9, F14, F16, F20): Sum = _______

**Avoidance Subscale** (items F5, F7, F8, F11, F12, F13, F17, F22): Sum = _______

**Hyperarousal Subscale** (items F2, F4, F10, F15, F18, F19, F21): Sum = _______

**Total IES-R Score** (sum of all 22 items): _______ (Range: 0-88)

**Interpretation:**

- Total score 0-32: Low stress
- Total score 33-38: Moderate stress
- Total score ≥39: Severe stress

**SECTION G: PANDEMIC-RELATED EXPERIENCES (QUANTITATIVE ASSESSMENT)**

**G1. Since the COVID-19 pandemic started, have you experienced increased social stigma or discrimination?**

1. ☐ No change
2. ☐ Slight increase
3. ☐ Moderate increase
4. ☐ Significant increase
5. ☐ Severe increase

**G2. Do people in your community confuse your TB symptoms (cough, fever) with COVID-19 symptoms?**

1. ☐ Never
2. ☐ Rarely
3. ☐ Sometimes
4. ☐ Often
5. ☐ Always

**G3. Have you experienced increased isolation from family members due to the pandemic?**

1. ☐ No change
2. ☐ Slight increase
3. ☐ Moderate increase
4. ☐ Significant increase
5. ☐ Severe isolation

**G4. Have you experienced difficulty accessing nutritious food recommended for TB treatment during the pandemic?**

1. ☐ No difficulty
2. ☐ Slight difficulty
3. ☐ Moderate difficulty
4. ☐ Significant difficulty
5. ☐ Severe difficulty (rarely access nutritious food)

**G5. How adequate was the government's response to support TB patients during the pandemic?**

1. ☐ Very adequate
2. ☐ Adequate
3. ☐ Neutral/Neither adequate nor inadequate
4. ☐ Inadequate
5. ☐ Very inadequate

**G6. During lockdowns, how often were you able to obtain your TB medications?**

1. ☐ Always when needed
2. ☐ Usually when needed
3. ☐ Sometimes faced delays
4. ☐ Often faced delays
5. ☐ Frequently unable to obtain medications

**G7. How has your stress level changed since the COVID-19 pandemic began?**

1. ☐ Significantly decreased
2. ☐ Slightly decreased
3. ☐ No change
4. ☐ Slightly increased
5. ☐ Significantly increased

**G8. How has your overall quality of life changed since the COVID-19 pandemic began?**

1. ☐ Significantly improved
2. ☐ Slightly improved
3. ☐ No change
4. ☐ Slightly worsened
5. ☐ Significantly worsened

**SECTION H: DATA QUALITY AND VERIFICATION**

**H1. Interview completed in:**

1. ☐ Bengali
2. ☐ English
3. ☐ Other language (specify): _______________

**H2. Interview location:**

1. ☐ TB treatment center
2. ☐ Participant's home
3. ☐ Other (specify): _______________

**H3. Was the participant able to understand and respond to all questions?**

1. ☐ Yes, all questions understood
2. ☐ Needed clarification on some questions
3. ☐ Significant difficulty understanding

**H4. Data collector assessment of participant's reliability:**

1. ☐ Very reliable
2. ☐ Generally reliable
3. ☐ Somewhat reliable
4. ☐ Questionable reliability

**H5. Were there any interruptions during the interview?**

1. ☐ No interruptions
2. ☐ Minor interruptions
3. ☐ Significant interruptions

**H6. Data collector notes/comments:**

**H7. Data collector signature:** ______________________ Date: __________

**H8. Supervisor review completed:**

Supervisor name: __________________________

Supervisor signature: ______________________ Date: __________

**H9. Data entry completed:**

Data entry operator: __________________________

Date entered: __________ Time: __________

**H10. Data quality check completed:**

Checked by: __________________________

Date checked: __________ Issues found: ☐ Yes ☐ No

**QUALITATIVE INTERVIEW GUIDE (FOR SUBSAMPLE n=36)**

**Note:** The following semi-structured interview guide was used for in-depth qualitative interviews with a purposively selected subsample of 36 participants. Interviews lasted 25-40 minutes and were audio-recorded with participant permission.

**Introduction**

*"Thank you for agreeing to this additional interview. I would like to understand more deeply your experiences during the COVID-19 pandemic as a person being treated for tuberculosis. There are no right or wrong answers. Please feel free to share your thoughts and experiences openly. Everything you say will be kept confidential."*

**Domain 1: Pandemic Perceptions and Attitudes**

1. What were your first thoughts and feelings when you heard about the COVID-19 pandemic?
2. How do you understand the relationship between TB and COVID-19? Do you think having TB makes you more vulnerable to COVID-19?
3. What do you think about the government restrictions (lockdowns, movement limitations) during the pandemic?
4. How did these restrictions affect you personally?

**Domain 2: Physical Health Impacts**

5. How has your physical health been during the COVID-19 pandemic compared to before?
6. Have you experienced any new symptoms or worsening of existing symptoms during the pandemic?
7. Tell me about your experience accessing TB treatment services during the pandemic. What challenges did you face?
8. How have the TB medications affected you during this time? Any changes in side effects or tolerance?

**Domain 3: Psychological and Emotional Impacts**

9. How has the pandemic affected your mental health and emotional well-being?
10. Can you describe your feelings when you have to cough in public during the pandemic? How do people react?
11. What are your biggest fears or worries during this time?
12. How do you cope with stress and anxiety?

**Domain 4: Social Impacts and Stigma**

13. How have your relationships with family members changed during the pandemic?
14. Tell me about any experiences of stigma or discrimination you have faced because of your TB diagnosis during the pandemic.
15. Do people in your community confuse TB symptoms with COVID-19 symptoms? Can you give examples?
16. How has your social life and interaction with friends and neighbors changed?
17. Have you felt isolated or alone? Tell me about that experience.

**Domain 5: Economic Impacts**

18. How has the pandemic affected your work and income?
19. What economic challenges have you faced in continuing TB treatment during the pandemic?
20. Can you describe the costs associated with traveling to the TB clinic? How has this changed during the pandemic?
21. How has the pandemic affected your ability to buy food, especially nutritious food recommended for TB treatment?
22. What financial coping strategies have you used during this time?

**Domain 6: Healthcare Access and Treatment Adherence**

23. Describe your typical journey to the TB treatment center before and during the pandemic. What changed?
24. Have you missed any clinic appointments during the pandemic? If yes, why?
25. How has the frequency of your clinic visits changed? How do you feel about this?
26. Have you experienced any interruptions in your TB medication supply? Tell me about that.
27. What support have you received from healthcare workers during the pandemic?

**Domain 7: Nutritional Sufficiency**

28. What does a typical meal look like for you now? How has this changed since the pandemic?
29. What foods do your doctors recommend for TB treatment? Are you able to eat these foods?
30. What barriers do you face in accessing nutritious food?

**Domain 8: Government Response and Support**

31. What assistance, if any, have you received from the government during the pandemic?
32. Do you think the government's support has been adequate for TB patients? Why or why not?
33. What specific support do you think TB patients need during pandemic situations?
34. If you were advising the government, what would you recommend they do to help TB patients during health emergencies?

**Domain 9: Proximity to COVID-19 Cases**

35. Do you know anyone who has been infected with COVID-19? How has this affected you?
36. Have you been tested for COVID-19? What was that experience like?
37. What precautions do you take to protect yourself from COVID-19 while continuing TB treatment?

**Domain 10: Coping Mechanisms and Resilience**

38. What gives you strength to continue your TB treatment during these difficult times?
39. Who or what has been your main source of support?
40. What strategies have helped you manage the challenges of having TB during the pandemic?
41. What advice would you give to other TB patients facing similar situations?

**Closing Questions**

42. Is there anything else you would like to share about your experience as a TB patient during the COVID-19 pandemic that we haven't discussed?
43. What is your greatest hope for the future?
44. Do you have any questions for me?

*"Thank you very much for sharing your experiences with me. Your insights are very valuable for improving support for TB patients during health emergencies."*

**INSTRUMENT CITATIONS AND VALIDATION**

**EQ-5D-3L**

**Original Source:** EuroQol Group (1990). EuroQol-a new facility for the measurement of health-related quality of life. *Health Policy*, 16(3), 199-208. <https://doi.org/10.1016/0168-8510(90)90421-9>

**Bengali Validation:** This study used the Bengali-validated version of EQ-5D-3L which has been validated for use in Bangladesh populations.

**Description:** Generic health-related quality of life instrument with five dimensions (mobility, self-care, usual activities, pain/discomfort, anxiety/depression), each with three levels (no problems, some problems, extreme problems), plus visual analogue scale (0-100).

**Completion Time:** Approximately 2-3 minutes

**Licensing:** EQ-5D is copyrighted by the EuroQol Group. Permission to use must be obtained from <https://euroqol.org/>

**Impact of Event Scale - Revised (IES-R)**

**Original Source:** Weiss, D. S., & Marmar, C. R. (1997). The Impact of Event Scale-Revised. In J. P. Wilson & T. M. Keane (Eds.), *Assessing psychological trauma and PTSD* (pp. 399-411). New York: Guilford Press.

**Further Reference:** Weiss, D. S. (2007). The Impact of Event Scale-Revised. In J. P. Wilson & C. S. Tang (Eds.), *Cross-cultural assessment of psychological trauma and PTSD* (pp. 219-238). Boston: Springer. <https://doi.org/10.1007/978-0-387-70990-1_10>

**Bengali Validation:** Bengali-validated version used in this study.

**Description:** 22-item self-report measure assessing subjective distress caused by traumatic events. Three subscales: Intrusion (8 items), Avoidance (8 items), Hyperarousal (6 items). Five-point scale (0-4). Total score 0-88.

**Scoring Interpretation:**

- 0-32: Low stress
- 33-38: Moderate stress
- ≥39: Severe stress

**Completion Time:** Approximately 5-7 minutes

**Sociodemographic and Clinical Questionnaire**

**Development:** Specifically developed for this study based on:

1. **Literature Review:** Extensive review of tuberculosis research, quality of life studies, and pandemic impact assessments
2. **Expert Consultation:** Input from TB clinicians at icddr,b, public health researchers at Jahangirnagar University, and epidemiologists with expertise in Bangladesh TB programs
3. **Pilot Testing:** Pilot tested with 10 TB patients not included in the final study sample to ensure:
   - Cultural appropriateness for Bangladesh context
   - Clarity of questions in Bengali translation
   - Appropriate response categories
   - Acceptable interview duration
   - Sensitive handling of potentially stigmatizing questions
4. **Reference Instruments:** Adapted selected items from validated TB research questionnaires while ensuring context-specific relevance for pandemic conditions

**Domains Covered:**

- Sociodemographic characteristics (13 items)
- TB clinical characteristics (18 items)
- COVID-19 pandemic impact (7 items)
- Pandemic-related experiences (8 items)
- Data quality assessment (10 items)

**Total Questionnaire Completion Time:** 30-45 minutes (including EQ-5D-3L and IES-R)

**END OF QUESTIONNAIRE**

**For additional information contact:**

**Principal Investigator:** Santu Das
Department of Economics
Jahangirnagar University
Savar, Dhaka 1342, Bangladesh
Email: [santudasbd7@gmail.com](mailto:santudasbd7@gmail.com)
ORCID: <https://orcid.org/0009-0008-2052-4756>
